# Differences in Metabolic Syndrome Prevalence by Employment Type and Sex

**DOI:** 10.3390/ijerph15091798

**Published:** 2018-08-21

**Authors:** Duk Youn Cho, Jung-Wan Koo

**Affiliations:** 1 Department of Rehabilitation and Assistive Technology, Korea National Rehabilitation Center, Seoul 01022, Korea; guylian77@gmail.com; 2 Department of Occupational and Environmental Medicine, Seoul St. Mary’s Hospital, College of Medicine, The Catholic University of Korea, Seoul 06591, Korea

**Keywords:** non-standard work, metabolic syndrome, Korean workers, socioeconomic status, sex

## Abstract

Workers may sometimes do the same work, but differ in their risk of health-related problems depending on whether the employment type is standard or non-standard. Furthermore, even with similar job and employment types, there may be differences in risk factors for health-related problems depending on sex. This study aimed to determine the prevalence of metabolic syndrome (MetS) by employment type and sex using data from the Fifth Korean National Health and Nutrition Examination Survey (KNHANES Ⅴ) (2010–2012) and KNHANES Ⅵ (2013–2015) conducted by the Korea Centers for Disease Control and Prevention. Overall, 9523 adult wage workers (5523 standard workers and 4000 non-standard workers) aged ≥ 19 years were analyzed. To determine MetS prevalence odds ratios according to employment type, logistic regression analysis was performed disaggregated by sex. The prevalence of MetS significantly increased with age (*p* < 0.001), being married (*p* < 0.05), current smoking status (*p* < 0.05), and high-risk drinking (*p* < 0.001) among male subjects. The prevalence of MetS significantly increased among female manual workers (*p* < 0.001), those with lower educational level and household income (*p* < 0.001). Non-standard workers of either sex showed higher MetS prevalence than standard workers; only females showed significant difference (*p* < 0.001). Female non-standard workers showed 1.44, 1.33, and 1.34 (all *p* < 0.001) times higher odds of MetS prevalence in Models 1, 2, and 3, respectively, compared to standard workers, suggesting a difference in risk factors of MetS according to sex. Also, that employment type affects MetS prevalence suggests that employment pattern is an important risk factor especially in females. Therefore, to manage MetS in female non-standard workers, individual health care as well as social effort may be necessary.

## 1. Introduction

Since the 2008 financial crisis, economic uncertainty has persisted throughout the world and this has resulted in continual job instability [1]. In Korea, unstable jobs such as non-standard employment began to emerge during the International Monetary Fund (IMF) economic crisis in 1997, and this trend has continued to this day [2]. Non-standard workers are mostly employed under working conditions with low wage levels and are treated differently regarding wages, including incentives and welfare benefits [3]. In addition, many non-standard workers have more than one job due to the low wages, and work during the weekend or late [4]. Long work hours, irregular lifestyle, and high risk of job stress exposure among non-standard workers seriously threatens their health and safety [4,5]. Long working hours or high occupational stress cause short-term problems such as stress, fatigue, lack of sleep, smoking, excessive drinking, and lack of exercise, leading to long-term problems such as digestive diseases, urogenital diseases, musculoskeletal diseases, and mental diseases [6]. The abovementioned short and long-term problems may individually threaten the health of workers, and various problems may be combined, resulting in chronic diseases.

Recently, the prevalence of metabolic syndrome (MetS) is rapidly increasing worldwide. The risk factors for MetS include aging, physical inactivity, Western diet, sedentary work, long working hours, and high occupational stress [7]. In other words, lifestyle and socioeconomic status are important risk factors for MetS [8]. The factors associated with MetS are closely related to cardiovascular risk factors or diabetes, including abdominal obesity, triglyceride levels, high-density lipoprotein (HDL) cholesterol levels, hypertension, and fasting plasma glucose levels [9]. Risk factors for MetS can be controlled and prevented through appropriate management. However, many non-standard workers are exposed to multiple risk factors regarding improper health management due to lack of money and time. In recent years, many studies have examined the differences among various occupational groups such as manual workers, non-manual workers, office workers, and firefighters concerning the effect of socioeconomic status on MetS [8,10,11,12]. In previous studies, there was no consensus on the association between job type and MetS prevalence. However, there was a consensus regarding a difference in risk factors affecting MetS prevalence according to sex [11,13,14]. However, even if they have the same occupation, health-related characteristics of workers may differ depending on whether the employment is standard or non-standard. Nevertheless, little research has been done on the difference in MetS prevalence according to employment type. Therefore, in this study, we aimed to determine the prevalence of MetS by employment type and by sex.

## 2. Materials and Methods 

### 2.1. Subjects

This study used raw data from the Fifth Korean National Health and Nutrition Examination Survey (KNHANES V) (2010–2012) and the KNHANES VI (2013–2015) conducted by the Korea Centers for Disease Control and Prevention. A stratified multistage cluster probability sampling design was used to select representative samples of denormalized Korean civilians. A trained investigator visited subjects’ homes directly for the standardized health evaluation and with a questionnaire. In order to assess the association between MetS and full-time employment, we used data regarding wage worker subjects aged 19 years or older with employment status. From 2010 to 2015, the final 9523 (5523 standard and 4000 non-standard) workers met the inclusion criteria, out of 48,482.

### 2.2. Definition of Variables

#### 2.2.1. Metabolic Syndrome

The definition of MetS was based on the National Cholesterol Education Program Adult Treatment Panel (NCEP ATP III) criteria, in which three or more of the following five risk factors were considered: (1) abdominal obesity(defined for Koreans as a waist circumference of ≥90 cm in males and ≥85 cm in females); (2) triglyceride level ≥ 150 mg/dL; (3) HDL cholesterol level < 40 mg/dL in males and <50 mg/dL in females; (4) systolic blood pressure ≥ 130 mmHg or diastolic blood pressure ≥ 85 mmHg; and (5) fasting plasma glucose level ≥ 110 mg/dL [15].

For the measurement of waist circumference, the subject was allowed to comfortably rest his/her arms on his/her waist while looking forward with his/her bare skin exposed. The lower end of the last palpable rib and the upper points of the iliac crest on both sides were measured in the subject’s midaxillary line. The subjects were asked to exhale; the tape was then pulled to such a point that the skin was not pressed, and measured to one decimal place. Fasting blood glucose, HDL cholesterol, and triglyceride levels were measured from blood samples. Blood pressure was measured using a mercury sphygmomanometer. Prior to blood pressure measurement, subjects rested for 5 min; then the blood pressure was measured once. They then rested for 30 s and the blood pressure was measured again. The average value was calculated from the two measurements. All measurements were made in accordance with the National Health and Nutrition Examination Guidelines.

#### 2.2.2. Job-Related Variables

In the sixth survey (2013–2015), we distinguished between standard and non-standard workers using the variable “whether standard worker (EC_Wht_0)”. However, no similar variable existed in the fifth survey (2010–2012) to confirm the standard worker status. Thus, standard and non-standard workers were distinguished using the variables “working hours (EC_WH)” and “employee status wage workers (EC_STT_2)”. Standard workers were defined as “full-time and regular” workers. Non-standard workers were defined as “full-time and temporary”, “full-time and daily”, “hourly and regular”, “hourly and temporary” or “hourly and daily workers” [16,17,18]. Non-manual and manual workers complied with the job classification of the Korean Standard Classification of Occupations [18,19]. Those classified as non-manual workers were managers, professionals, technicians, sales, and service workers. Those classified as manual workers were those engaged in agriculture, forestry, fishery, manufacturing, construction, mining and soldiers.

#### 2.2.3. Lifestyle-Related Variables

Smoking status was classified as current smoker, ex-smoker, and non-smoker. Ex-smoker referred to a person who had smoked in the past but did not currently smoke, and a non-smoker referred to a person who had never smoked. Drinking was classified into high risk, moderate, or non-drinking. In Korea, high-risk drinking is defined as drinking more than 13 standard servings for men and over six standard servings for women on a weekly basis [20]. High-risk drinking was used in reference to males and females who drank more than 2–3 times in a week with more than 7–9 servings at a time and more than 5–6 servings, respectively, at a time. In 2014 and 2015, physical activity was confirmed using the practice rate of “aerobic physical activity”. In the data before 2013, there was no variable for the practice rate of “aerobic physical activity”, “high-intensity physical activity time” and “moderate physical activity time” were calculated according to the guideline on “aerobic physical activity practice rate”. The “aerobic physical activity rate” guideline set the moderate physical activity for a week at more than 150 min, or the high-intensity physical activity at 75 min or more, or the previous two physical activities for a considerable amount of time. The denominator was set as the total number of subjects aged 19 years or older. High-intensity physical activity at 1 min was calculated as 2 min of moderate physical activity. Body mass index value was derived from the “anthropometric measurements”. Stress was confirmed in four stages: “I feel stressed very much”, “I feel stressed a lot”, “I feel stressed a little” and “I rarely feel stressed”. Depression was identified as a current disease.

#### 2.2.4. Sociodemographic Characteristics

The sociodemographic characteristics of the subjects included age, educational level, marital status, and household income level. Educational level is divided into “elementary school”, “middle school”, “high school” and “≥university”. Marital status was classified into “married” if “married” or “living together” but as “others” regardless of the reason, if not living together. Household income level was divided into four stages from the first to the fourth quartiles. Data regarding the area of residence were identified in the results from Seoul to Jeju.

### 2.3. Statistical Analysis

In this study, a composite sample analysis was performed using raw data from the National Health and Nutrition Examination Survey, which was extracted using a two-stage stratified sampling design, rather than a simple random sample design. Stratification and cluster variables were applied and the composite sample design was conducted by applying the questionnaire survey weight. Frequency analysis was performed to report on the general characteristics and major variables of the study subjects. Chi-square analysis was used to determine the prevalence of MetS according to the general characteristics and MetS prevalence according to full-time employment. Odds ratios (ORs) were calculated by logistic regression analysis to determine the effects of age, lifestyle, and job-related factors on the prevalence of MetS. All statistical analyses were conducted using SPSS version 22.0 (IBM corp., Armonk, NY, USA). A *p* value < 0.05 was considered statistically significant. The study protocol was approved by the institutional review board of the Catholic University of Korea, College of Medicine (approval ID: KC18ZESI0410).

## 3. Results

### 3.1. General Characteristics of Subjects Included in the Study to Determine Metabolic Syndrome Prevalence

The study subjects were wage workers aged 19 years and older including 5006 men (52.6%), and 4517 women (47.4%), totaling 9523 workers. The prevalence of MetS differed significantly according to age and sex and seemed to increase significantly, for both sexes, with increasing age (*p* < 0.001). However, aside from age, other variables with significant differences in MetS prevalence differed between male and female workers (Table 1). 

Males showed significant differences in MetS prevalence in lifestyle-related variables such as smoking, drinking, and marital status. Among male subjects, MetS prevalence was significantly higher among current smokers (*p* < 0.05), the high-risk drinking group (*p* < 0.001), and among the married (*p* < 0.05). On the other hand, among female subjects, there were differences in socioeconomic variables. Lower educational level (*p* < 0.001), household income (*p* < 0.001), and manual work (*p* < 0.001) were related to a higher prevalence of MetS.

### 3.2. Metabolic Syndrome Prevalence According to Sex and by the Standard of Work

Among the male subjects, 3561 (71.1%) were standard workers and 1445 (28.9%) were non-standard workers. The prevalences of MetS among standard and non-standard workers were 26.1% and 28.2%, respectively, and the proportion of non-standard workers was high. However, there was no significant difference between the two groups. Among female subjects, 1962 (43.4%) were standard workers and 2555 (56.4%) were non-standard workers. The prevalences of MetS among the female subjects were 13.5% and 25.8% for standard and non-standard workers, respectively. Non-standard workers showed a statistically significant difference in MetS prevalence compared to standard workers (*p* < 0.001) (Table 2).

### 3.3. Factors Affecting Metabolic Syndrome

To determine the factors affecting MetS, logistic regression analysis was performed. The model consisted of three stages. Models 1, 2, and 3were adjusted for age, lifestyle factors, and factors related to economic activity, respectively. Regression analysis among male subjects showed that non-standard employment status in all models did not increase MetS prevalence. Among female non-standard workers, however, there was a 1.44 times increase in the risk of MetS in model 1 (OR = 1.44, *p* < 0.001), 1.33 times increase in risk in model 2 (OR = 1.33, *p* < 0.001), and 1.34 times increase in the risk in model 3 (OR = 1.34, *p* < 0.001) (Table 3).

## 4. Discussion

In recent studies, lifestyle-related factors such as age, unhealthy diet, and sedentary lifestyle, as well as socioeconomic status and environmental factors such as job type, occupational stress, and working hours have been reported as important causes of the increasing MetS prevalence [7,8,21,22]. In this study, we analyzed the differences in MetS prevalence with emphasis on employment type among the socioeconomic factors. As a result, MetS prevalence was shown among non-standard workers to be higher than that among standard workers. In particular, female subjects had a 1.34-fold higher risk of MetS prevalence among non-standard workers than among standard workers. This is consistent with the results of a study in which the risk of hypertension in non-standard female workers was 1.42 times higher than that of standard workers due to employment type and sex differences in cardiovascular health [18]. Hypertension is one of the risk factors for MetS, and might have affected the increased risk of MetS in female non-standard workers. The results of this study showed that female workers were more affected by socioeconomic factors than male workers. Among female workers, the lower the educational and household income levels, the higher the prevalence of MetS. Low educational and household income levels could cause differences in opportunities for workers to access health services and affect health-related behaviors [23,24]. In particular, the lower the socioeconomic status of female workers, the less they care about health care and hence, the higher the risk of MetS [12]. As a result of MetS prevalence according to job type, the prevalence among manual workers was higher than that among non-manual workers [25]. The highest MetS prevalence occurred among female skilled workers in the agricultural and fishing industries. MetS prevalence was higher among low-income and local residents [26,27]. These non-standard workers have low socioeconomic status and are treated poorly in wages, working conditions, and social safety [28]. Non-standard workers have limited working periods, resulting in unstable occupation that reduces perceived health condition, increases psychological stress, and affects physical health [29]. Psychological stress and physical health problems in non-standard workers increase the risk of chronic and acute diseases; furthermore, the social, physiological, and self-rated health of workers become lowered [30]. Finally, the difference in socioeconomic status due to unstable employment is a factor for health deterioration and social determinants of health [1]. Problems associated with low socioeconomic status among these non-standard workers have a greater impact on female workers than male workers, which ultimately increases the prevalence of MetS among female non-standard workers.

On the other hand, male workers showed no differences in economic level, educational level, and job type, and significant differences occurred in MetS prevalence among factors related to lifestyles such as smoking and drinking. Smoking increases the risks of low HDL cholesterol level, higher triglyceride level, and abdominal obesity, thus increasing the prevalence of MetS among male subjects [7]. Excessive drinking increases the risk of low HDL cholesterol level and cohort studies among Korean office workers over the past decade have also identified male sex as a risk factor for MetS [7,11]. Thus, unhealthy lifestyles such as smoking and drinking are closely related to the risk factors for MetS [31]. However, with smoking and drinking adjusted for, the two variables failed to show an increase in the prevalence of MetS among male non-standard workers. In other words, unhealthy lifestyle has a greater impact on MetS prevalence than differences in socioeconomic status. Thus, the inverse correlation between socioeconomic status and MetS in males was confirmed in previous studies [14,25,32]. Among males, non-manual workers with high levels of education and income had the highest risk of MetS, and the higher the socioeconomic status, the higher the risk of MetS [25,32]. In addition, male subjects were less likely to have MetS among workers with higher levels of physical activity [14]. The high prevalence of MetS among office workers is attributed to their long working hours, longer sitting periods, and more exposure to risk factors that can lead to MetS. Moreover, male office workers are known to have more opportunities for drinking [12,27,33,34,35]. A study of firefighters showed that men tend to enjoy healthier lifestyles with shift work and stressful job conditions, and that this lifestyle resulted in a lower prevalence of MetS among them than office workers [36]. These results suggest that male workers have higher socioeconomic status than female workers, and that the type of work and their lifestyle are important factors for MetS prevalence. Similar to this study, previous studies have reported sex differences in the risk factors for MetS [11,13,25,37,38]. Hypertension, hyperglycemia, and high triglyceride level were major risk factors for MetS in males, and obesity and low HDL cholesterol level were the most common risk factors for females [13]. 

There are three possible reasons for the differences in MetS prevalence between male and female non-standard workers, based on previous studies on the difference in risk factors for MetS according to sex. The first is that female non-standard workers are more affected by work-related psychosocial factors due to lower socioeconomic classes. The proportion of non-standard workers among female workers is higher than that of non-standard male workers among male workers. In this study, 28.8% of males and 56.6% of females were classified as standard and non-standard workers from 2010 to 2015. Females often work as administrative support workers rather than managers, and often quit because of marriage, pregnancy, and childcare [11]. In addition, even after re-employment, it is difficult to resume in stable jobs because of the long leave period. For this reason, many females work as non-standard workers, and as temporary or part-time workers. With this low socioeconomic status, female workers are easily exposed to job-related psychological stress and eventually to MetS risk factors [25]. Female non-standard workers show poor mental health status such as high depression and suicidal ideation levels [39]. In addition, the ability to overcome psychological stress was lower than that among men [25,33]. Meta-analysis of the effects of work-related psychosocial factors on MetS revealed significant risk factors for some components associated with MetS such as weight gain, obesity, and hypertension [40].

The second reason is the high risk of obesity in female non-standard workers. Several studies have reported that the risk of obesity in female non-standard workers is higher than that in male non-standard workers [13,37]. Obesity and insulin resistance play a key role in the development of MetS [18,41]. Obesity increases the amount of free fatty acids in the body, which in turn increases insulin resistance, leading to cardiovascular diseases such as diabetes, lipid abnormalities, and hypertension [37]. Ultimately, the high risk of obesity affects the risk of cardiovascular disease and increases the risk of MetS [14].

The third reason is due to the physiological characteristics of female workers. In Korea, about 30% of female workers are mostly pink-collar workers such as nurses, stewardesses, nannies, babysitters, hairdressers, and administrative assistants [13]. These pink-collar workers engage more in shift work, and this results in a 5% higher prevalence of menstrual irregularity than among daily workers [6]. Females with menstrual irregularities are often diagnosed with polycystic ovary syndrome, and such females have a high risk of MetS [6]. However, the increased prevalence of MetS due to these hormonal problems may be due to shift work, which may be difficult to identify among differences in standard and non-standard workers. Nonetheless, the risk of MetS in non-standard workers is higher because they are unable to take immediate action concerning their problems, even if it occurs while doing the same thing [5]. These results suggest that the prevalence of MetS among female non-standard workers was higher than that among standard workers. Psychological problems caused by differences in socioeconomic status increase the risk of MetS in female non-standard workers, and the combination of physiological characteristics of females and that of being a non-standard worker lead to anxiety and consequent poor treatment.

The limitations of this study are as follows: First, the cross-sectional data used in the study may not clearly show a causal relationship between factors. Second, there might be information bias due to the self-reported questionnaire. For example, responses to smoking among female subjects may not be representative. Finally, the definitions of standard and non-standard work varied according to the survey period. In the sixth survey conducted between 2013 and 2015, there was a question to directly confirm standard and non-standard (EC_Wht_0) work, but there was no such question in the fifth survey conducted between 2010 and 2012. Therefore, the two items “job status wage worker (EC_STT_2)” and “working hours (EC_WH)” were defined according to non-standard worker standards. However, international standards for non-standard workers are not clear and there is a limit to how these apply in all countries [18]. Despite these limitations, this sample is representative of the results of the six-year data systematically surveyed in the entire Republic of Korea.

In this study, the prevalence of MetS in female non-standard worker was increased, and it was confirmed that the difference in socioeconomic status according to gender affects MetS prevalence. Thus, a fundamental change in employment patterns will be needed to address health-related problems that arise from non-standard work. The recent policy of converting non-standard work to standard work by the government will be a way to overcome the difference in health risk due to working style. However, there are difficulties in policy promotion due to various interests including current salary level, welfare problem, and reverse discrimination against standard workers. Employment stability and work environment improvement for non-standard workers are not only a matter of interest in payroll improvement but also an important solution to lower the social cost of health care and improve the quality of life and health care of all citizens. It is expected that the results of this study, showing the high risk of MetS in non-standard workers could be utilized as a basis for government policy promotion on health.

## 5. Conclusions

The results of this study confirm that the prevalence of MetS among non-standard female workers increased according to employment type. In addition, the risk factors leading to increases in the prevalence of MetS differed according to sex. In the future, direct research and long-term observation of psychosocial, lifestyle, and biological factors among non-standard workers will be needed using a cohort study of non-standard workers. It is necessary to clarify the cause of high MetS prevalence among female non-standard workers. In addition, among the factors affecting MetS, it is considered that lifestyle-related problems need to be investigated since these did not result in increased MetS prevalence among male non-standard workers.

## Figures and Tables

**Table 1:** General characteristics of subjects included in the study to determine metabolic syndrome prevalence (*N* = 9523).

Characteristics	Metabolic Syndrome, *N* (%)					
	Men (*n* = 5006)			Women (*n* = 4517)		
	Yes	No	*p*	Yes	No	*p*
Age (years)			<0.001			<0.001
19–29	35 (14.9)	211 (85.1)		14 (5.3)	315 (94.7)	
30–39	288 (21.9)	1038 (78.1)		79 (17.2)	896 (90.9)	
40–49	406 (28.8)	1035 (71.2)		202 (17.2)	1079 (82.8)	
50–59	364 (33.7)	752 (66.3)		322 (28.1)	816 (71.9)	
60–69	196 (29.9)	478 (70.1)		245 (45.0)	300 (55.0)	
≥70	53 (23.9)	160 (76.1)		118 (48.0)	131 (52.0)	
Education level			0.073			<0.001
≤Elementary school	115 (28.0)	331 (72.0)		398 (41.0)	544 (59.0)	
Middle school	147 (32.6)	306 (67.4)		160 (29.2)	368 (70.8)	
High school	461 (26.6)	1212 (73.4)		303 (18.4)	1322 (81.6)	
≥University	619 (25.5)	1815 (74.5)		119 (8.7)	1303 (91.3)	
Job type			0.244			<0.001
Non-manual worker	766 (27.4)	2010 (72.6)		454(15.3)	2388(84.7)	
Manual worker	576 (25.7)	1654 (74.3)		526(29.6)	1149(70.4)	
Marital status			0.019			0.271
Married	1247 (27.3)	3340 (72.7)		736 (19.9)	2810 (80.1)	
Others	95 (21.0)	324 (79.0)		244 (21.8)	727 (78.2)	
Household income level			0.386			<0.001
1st quartile	474 (27.7)	1221 (72.3)		208 (14.9)	1170 (85.1)	
2nd quartile	435 (26.4)	1270 (73.6)		265 (18.8)	1117 (81.2)	
3rd quartile	331 (25.0)	931 (75.0)		285 (22.1)	862 (77.9)	
4th quartile	102 (29.5)	242 (70.5)		222 (34.5)	388 (65.5)	
Smoking			0.003			0.273
Current smoker	596 (29.0)	1524 (71.0)		61 (22.4)	201 (77.6)	
Ex-smoker	516 (25.9)	1394 (74.1)		36 (15.7)	166 (84.3)	
Non-smoker	230 (22.6)	746 (77.4)		883 (20.4)	3170 (79.6)	
Drinking			<0.001			0.882
High risk drinking	365 (34.2)	711 (65.8)		43 (20.2)	170 (79.8)	
Moderate drinking or non-drinking	977 (24.4)	2953 (75.6)		937 (21.7)	3367 (78.3)	
Physical activity			0.261			0.111
Yes	613 (25.8)	1680 (74.2)		336 (21.9)	1151 (78.1)	
No	729 (27.4)	1984 (72.6)		644 (19.5)	2386 (80.5)	

Data were analyzed using Pearson’s χ^2^ test, *p* < 0.05.

**Table 2:** Metabolic syndrome prevalence according to sex and by the standard of work.

Sex	Employment Type	Metabolic Syndrome, *N* (%)		Total	*p*
		Yes	No		
Male	Standard workers	942 (26.4)	2619 (73.6)	3561 (100.0)	0.174
	Non-standard workers	400 (28.2)	1045 (71.8)	1445 (100.0)	
	Total	1342 (26.6)	3664 (73.4)	5006 (100.0)	
Female	Standard workers	271 (13.5)	1691 (86.5)	1962 (100.0)	<0.001
	Non-standard workers	709 (25.8)	1846 (74.2)	2555 (100.0)	
	Total	980 (20.3)	3537 (79.7)	4517 (100.0)	

Data were analyzed using Pearson’s *χ*^2^ test, *p* < 0.05.

**Table 3:** Prevalence odds ratios of factors affecting metabolic syndrome.

Sex	Model1 *		Model 2 **		Model 3 ***	
	Standard Workers	Non-Standard Workers	Standard Workers	Non-Standard Workers	Standard Workers	Non-Standard Workers
Males	Reference	0.97 (0.82–1.15)	Reference	0.94 (0.78–1.15)	Reference	0.97 (0.78–1.20)
Females	Reference	1.44 (1.18–1.77)	Reference	1.33 (1.07–1.65)	Reference	1.34 (1.07–1.67)

* adjusted for age. ** adjusted for age, smoking, drinking, physical activity, BMI, stress, and depression. *** adjusted for age, smoking, drinking, physical activity, BMI, stress, depression, household income, education level, job type, marital status, and region. BMI, body mass index.
